# Comparing the haemodynamic effects of high- and low-dose opioid anaesthesia: a secondary analysis of a randomised controlled trial

**DOI:** 10.1007/s10877-024-01195-6

**Published:** 2024-07-20

**Authors:** O. M. Marges, J. P. Nieboer, I. N. de Keijzer, R. Rettab, K. van Amsterdam, T. W.L. Scheeren, A. R.A. Absalom, H. E.M. Vereecke, M. M.R.F. Struys, J. J. Vos, J. P. van den Berg

**Affiliations:** 1 Department of Anaesthesiology, University Medical Centre Groningen, Groningen, Netherlands; 2 Department of Anaesthesia and Reanimation, AZ Sint-Jan Brugge AV, Bruges, Belgium; 3 Department of Anaesthesia and Peri-Operative Medicine, Ghent University, Gent, Belgium

**Keywords:** General anaesthesia, Induction, Hypotension, Pharmacology, Haemodynamics

## Abstract

Post-induction hypotension (MAP < 65 mmHg) occurs frequently and is usually caused by the cardiovascular adverse effects of the anaesthetic induction drugs used. We hypothesize that a clinically significant difference in the incidence and severity of hypotension will be found when different doses of propofol and remifentanil are used for induction of anaesthesia. Methods: This is a secondary analysis of a randomised controlled trial wherein four groups (A-D) of patients received one out of four different combinations of propofol and remifentanil, titrated to a predicted equipotency in probability of tolerance to laryngoscopy (PTOL) according to the Bouillon interaction model. In group A, a high dose of propofol and a low dose of remifentanil was administered, and across the groups this ratio was gradually changed until it was reversed in group D. Mean and systolic arterial blood pressure (MAP, SAP) were compared at four time points (T_baseline_, T_post−bolus_, T_3min_, T_nadir_) within and between groups Heart rate, bispectral index (BIS) and the incidence of hypotension were compared. Results: Data from 76 patients was used. At T_post−bolus_ a statistically significant lower MAP and SAP was found in group A versus D (*p* = 0.011 and *p* = 0.002). A significant higher heart rate was found at T_3min_ and T_nadir_ between groups A and B when compared to groups C and D (p = < 0.001 and *p* = 0.002). A significant difference in BIS value was found over all groups at T_3min_ and T_nadir_ (both *p* < 0.001). All other outcomes did not differ significantly between groups. Conclusion: Induction of anaesthesia with different predicted equipotent combinations of propofol and remifentanil did result in statistically different but clinically irrelevant differences in haemodynamic endpoints during induction of anaesthesia. Our study could not identify preferable drug combinations that decrease the risk for hypotension after induction, although they all yield a similar predicted PTOL.

## Introduction

For anaesthetists, induction of general anaesthesia is a balancing act. On one hand they strive towards an early and adequate depth of anaesthesia, while on the other hand avoid the haemodynamic side effects caused by these drugs. Nonetheless, post-induction hypotension is seen in up to 18% of patients undergoing general anaesthesia [1]. Moreover, post-induction hypotension accounts for about 50% of the cumulative intraoperative hypotension observed [1, 2]. Intraoperative hypotension is associated with postoperative myocardial infarction, acute kidney injury, and increased mortality [3, 4].A common believe among anaesthesiologists is that hypnotics such as propofol cause more haemodynamic instability than opioids and that therefore a combination of high-dose opioids together with low-dose hypnotics would have a favourable effect on haemodynamic stability. Solid evidence for these assumptions remains scarce [5–7].

Previously, using effect-compartment target-controlled infusions and the predictions of probability of tolerance of laryngoscopy (PTOL) according to the Bouillon interaction model, we assessed the haemodynamic effects of different combinations of propofol and remifentanil target effect-site concentrations, all yielding a predicted PTOL of 90%, in a population of American Society of Anesthesiologists (ASA) physical status of I to III without severe cardiovascular comorbidities and undergoing neuro- or maxillofacial surgery. We defined these as Ce_T PROP_ and Ce_T REMI,_ and compared the effects **after** these infusions had reached a pharmacological pseudo-steady-state, by maintaining an equilibration time of at least 11 min after the start of infusion [7]. However, in clinical practice it is rarely acceptable to wait for 11 min in order to reach predictable effects and so this methodology is only applicable for scientific purposes. In clinical practice the hypotensive effect of both medications is most notable observed soon after induction, particularly as a bolus is initially administered to achieve rapid onset of anaesthesia [8]. Therefore, this secondary analysis aims to compare the magnitude of hypotension associated with an induction towards four predicted equipotent combinations of Ce_T, PROP_ and Ce_T, REMI_. We hypothesise that a clinically significant difference in haemodynamic changes and less post-induction hypotension will be found between the different combinations of Ce_T, PROP_ and Ce_T, REMI_.

## Materials and methods

This was a secondary analysis of a randomised controlled trial on the haemodynamic effects of different predicted equipotent combinations of Ce_T PROP_ and Ce_T REMI_ according to the Bouillon interaction model [7]. The original trial was approved by the local medical ethics committee (University Medical Centre Groningen, University of Groningen, Groningen, the Netherlands, METc 2013/267) and was registered at clinicaltrials.gov (#NCT02067936). Prior to enrolment written informed consent was obtained from all participants. The study was conducted in line with the principles of the Declaration of Helsinki. The data are reported according to the CONSORT statement [9]. For an in-detail description of the study subjects and procedures we refer to the original article [7].

### Population

Patients undergoing elective neurosurgical or maxillofacial surgery under general anaesthesia with an ASA 1–3 classification were included. Exclusion criteria were: BMI > 35 kg m^− 2^, central nervous system disorders, hepatic disease, use of alpha-antagonists, beta-blockers or medication that affects the central nervous system, overt signs of alcohol or drug abuse and contra-indications for the use of propofol or remifentanil. Patients were randomised into four groups of 20 participants each.

### Study procedures

**Fig. 1:**
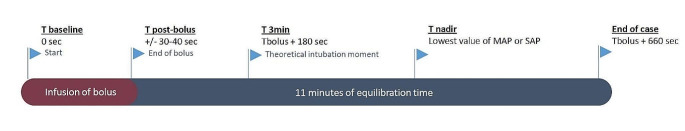
Timeline depicting the different time points during the study session

A detailed version of the study procedure can be found in our previous publication [7]. In brief, all patients were fastened for at least 6 h pre-operatively. They received an IV line which was connected to a bag of 500 ml balanced crystalloid fluid (Ringers Lactate 500ml) which ran in open flow during induction. No patient received more than 500 ml of fluids throughout the measurements. Four target effect-site propofol and remifentanil concentration combinations were chosen, using the predicted PTOL = 90% according to the Bouillon interaction model [10]. In group A, the effect site of propofol (Ce_T, PROP_) was set at 8.6 µg mL^− 1^, while that of remifentanil (Ce_T, REMI_) was set at 1.0 ng mL^− 1^. For groups B, C, and D, this was set respectively at 5.9 µg mL^− 1^ and 2 ng mL^− 1^, 3.6 µg mL^− 1^ and 4 ng mL^− 1^, and 2.0 µg mL^− 1^ and 8 ng mL^− 1^. Bispectral index (BIS, BIS™ Brain monitor, Medtronic, Minneapolis, MN, USA)) monitoring was applied as a surrogate measure of the hypnotic cerebral effect evoked by the administered drug combination. On the left middle finger of the patient, a continuous non-invasive blood pressure tool, Nexfin (formerly BMEYE, Amsterdam, Netherlands, now ClearSight^®^, Edwards Lifesciences, Irvine, USA), was attached for continuous blood pressure measurements. Subsequently, the propofol and remifentanil infusions were started simultaneously to achieve and maintain the predetermined effect-site concentrations using the Schnider model for propofol [11, 12] and the Minto model for remifentanil [13, 14]. The observation period started at the start of the infusion and ended after 11 min. Atropine 0.5 mg was administered if bradycardia (heart rate below 40 per min) occurred. If the mean arterial pressure (MAP) dropped below 50 mmHg, 5 mg of ephedrine was administered. The number of rescue interventions and the total amount of ephedrine and atropine were registered and compared in the original paper [7]. Both the patient and the anaesthetist in charge were blinded to the drug combination used. The moment directly before the start of propofol and remifentanil infusion was considered ‘baseline’ (T_baseline_). T_post−bolus_ was the moment directly *after* the end of the administration of the first bolus of propofol and remifentanil by the TCI pump. As the induction boli are administered by rapid infusion, T_post−bolus_ was ≈ 30–40 s after T_baseline_. For the third time point, T_3min_ was set at 3 min after the start of drug administration and was considered to be the moment when endotracheal intubation would usually be performed (the actual intubation was performed after 11 min, for the original study). T_nadir_ was defined as the time point when systolic blood pressure (SAP) and MAP were at their lowest within the study period: T_nadir_ could be a different timepoint for SAP (T_nadirSAP_) and MAP (T_nadirMAP_). If so, T_nadirMAP_ was used as T_nadir_, except for analyses of SAP. If ephedrine was given during the monitoring period, then only data acquired before ephedrine administration was used to determine T_nadir_. Figure 1 gives an overview of the different time points.

Data were collected by an unblinded researcher operating a laptop running RUGLOOP II software (DEMED engineering, Temse, Belgium) for data collection and controlling the target-controlled infusion pumps via which the study medication was administered. Data were extracted using R (The R Foundation for Statistical Computing, Vienna, Austria). R was also used to apply an artefact filter which removed all arterial blood pressure (ABP) values which differed more than 30% from the previous value. A 10 s moving median smoothing function (i.e. over a window from − 5 to + 5 s) was applied to all continuous variables. The remaining artefacts were removed using the following criteria commonly used in research regarding ABP measurements [2]:

1. SAP ≥ 300 mmHg, ≤ 20 mmHg or.
2. SAP ≤ diastolic arterial pressure (DAP) + 5 mmHg;
3. DAP ≤ 5 mmHg;
4. MAP ≥ SAP;
5. MAP < DAP.

Abrupt changes in SAP, defined as a change greater than or equal to 80 mmHg in either direction within one minute were also defined as artefacts and therefore removed.

### Outcomes

The primary outcome of this analysis was the incidence of hypotension. Hypotension was defined as a MAP < 65 mmHg or a SAP < 100 mmHg. These cut-off values were chosen according to the consensus of the Perioperative Quality Initiative (POQI) [15]. The difference between all relevant time points was calculated for SAP, as well as MAP. Additionally, the combination of the temporal and absolute change in ABP was quantified by calculating the differentials (in mmHg sec^− 1^) of ABP using the following formulas:

$$\:differential\:MAP=\frac{MAP\:or\:at\:T2-MAP\:at\:T1}{T2-T1}$$

$$\:differential\:SAP=\frac{SAP\:at\:T2-SAP\:at\:T1}{T2-T1}$$

The differentials between T_post−bolus_ and T_nadir_ and between T_3min_ and T_nadir_ were not calculated if T_nadir_ occurred before T_post−bolus_ or T_3min_ respectively. Other outcomes were the incidence of hypotension and the time until the first episode of hypotension. The time it took to reach the nadir in ABP (T_nadirSAP_ and T_nadirMAP_) was also compared between groups. Furthermore, heart rate and BIS values were compared between groups at the different time points.

### Statistical analysis

Categorical data are presented as numbers and percentages. Continuous data were assessed for normality using the Shapiro-Wilk test. The data were not normally distributed and therefore continuous data were presented as medians and interquartile ranges (IQR). A Kruskal-Wallis test was performed to compare continuous data between groups. A p-value < 0.05 was considered statistically significant. The Bonferroni correction was used to correct for inflation of Type I error rate in doing multiple corrections, which resulted in an adjusted p-value of 0.016. When a statistically significant difference was found, post hoc pairwise comparison using the Mann-Whitney U test was performed to determine which groups differed from each other. All statistical analyses were performed using SPSS (Version 23, IBM corporation). When more than 50% of the data was missing or when no data was available at baseline, the subject was excluded. Remaining missing data was accepted if it was incidental.

## Results

**Fig. 2:**
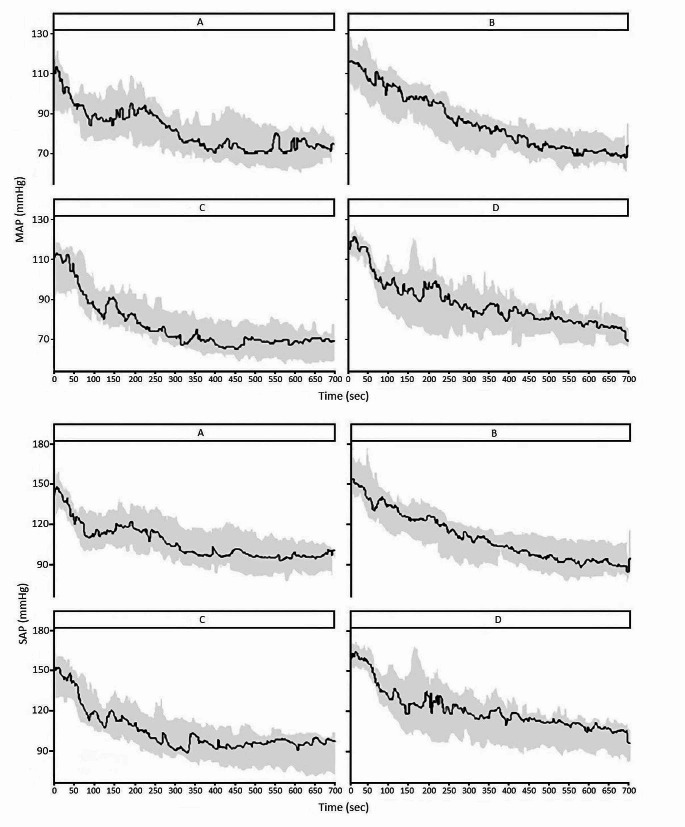
The evolution of MAP and SAP per group, from the start of medication until 11 min. The black line represents the mean MAP or SAP over time, the grey area represents the 95% confidence interval

In this secondary analysis, 4 patients (3 and 1 in groups C and D respectively) were removed from the data analysis because no ABP data was recorded at baseline (*n* = 3) or > 50% of ABP data was missing within the recorded timeframe (*n* = 1). Patient characteristics were comparable between groups and can be found in Table 1. More characteristics can be found in the original article [7]. Figure 2 shows a visual representation of the evolution of MAP and SAP per group from the start of infusion until 11 min.

Baseline ABP values did not differ significantly between groups. At T_post−bolus_ a significant higher MAP and SAP was found for group A versus D, *p* = 0.011 and p 0.002, respectively. MAP and SAP did not differ significantly between groups at any other time points as can be seen in Table 2. The lowest recorded MAP was comparable for groups A, B, C and D, respectively 62 [55–72] mmHg, 61 [54–73] mmHg, 57 [54–71] mmHg, and 66 [59–75] mmHg (*p* = 0.514). The lowest recorded SAP for groups A, B, C, and D were 83 [71–97] mmHg, 82 [66–95], 77 [69–94] mmHg, 85 [75–106] mmHg respectively, without reaching statistical significance (*p* = 0.575). The time it took to reach the lowest MAP/SAP value (T_nadir_) was 559 s [424–633], 559 s [388–644], 521 s [387–635], and 624 [514–672] seconds for groups A, B, C, and D, respectively without reaching statistical significance (*p* = 0.290 and *p* = 0.127 for MAP and SAP, respectively).

**Table 1:** Demographics

	Group A (*n* = 20)	Group B (*n* = 20)	Group C (*n* = 17)	Group D (*n* = 19)
Age (years)	50 (± 13)	52 (± 13)	48 (± 18)	57 (± 14)
Gender (m/f)	10/10	10/10	8/9	12/7
BMI	26 (± 3.99)	25 (± 3.19)	25 (± 5.48)	25 (± 5.43)

**Table 2:** Median absolute values of mean arterial pressure (MAP) and systolic arterial pressure (SAP) in mmHg with interquartile ranges at the different timepoint. At T_post−bolus_, regarding both MAP and SAP, only groups a and D differed significantly from each other

		Group A	Group B	Group C	Group D	*p*-value
MAP	T_baseline_	109 [91-117]	116 [104-127]	111 [92-121]	116 [109-122]	0.282
	T_post-bolus_	100 [90-109]	112 [98-127]	104 [93-115]	116 [107-117]	**0.011**
	T_3min_	89 [74-102]	96 [80-102]	76 [71-92]	96 [72-106]	0.234
	T_nadir_	62 [54-73]	61 [54-74]	57 [54-71]	66 [59-75]	0.514
SAP	T_baseline_	142 [125-162]	151 [138-169]	151 [129-166]	160 [146-170]	0.192
	T_post-bolus_	135 [123-144]	147 [133-169]	142 [125-153]	158 [151-167]	**0.002**
	T_3min_	116 [95-136]	123 [104-133]	104 [89-120]	128 [95-150]	0.297
	T_nadir_	82 [70-97]	82 [65-98]	77 [69-95]	85 [75-106]	0.575

The difference in MAP and SAP was calculated between all time points and no significant differences were found (Table 3). To incorporate the factor of time into these delta’s, differentials were calculated, without showing statistical significant difference between groups (Table 3).

Hypotension occurred in 59 of 76 subjects (77.6%). It occurred in 17 subjects (85%), 15 subjects (75%), 15 subjects (88.2%) and 12 subjects (63.2%) for groups A, B, C, and D respectively, and did not differ significantly (*p* = 0.251). The time until hypotension was 174 [62–312], 225 [104–410], 236 [114–368], and 206 [122–395] seconds for groups A, B, C, and D, respectively, and did not significantly differ between groups (*p* = 0.675).

**Table 3:** Median Δ and calculated median vectors of mean arterial pressure (MAP) and of systolic arterial pressure (SAP) in mmHg with interquartile ranges between the different timepoints; T_baseline_, T_bolus_, and T_nadir_ were respectively shortened to T_base_, T_bol_, and T_nadir_

		Group A	Group B	Group C	Group D	*p*-value
Δ MAP	T_base_-T_bol_	-7 [-13 - 3]	-2 [-7 - 4]	-6 [-9 - 0]	0 [-6 - 4]	0.302
	T_base_-T_3min_	-17 [-29 - -8]	-21 [-34 - -15]	-30 [-37 - -19]	-17 [-35 - -11]	0.274
	T_base_-T_nadir_	-43 [-54 - -34]	-52 [-63 - -40]	-48 [-58 - -37]	-46 [-56 - -36]	0.261
	T_bol_- T_3min_	-13 [- 24 - -2]	-21 [-34 - -12]	-26 [-36 - -10]	-17 [-35 - -5]	0.333
	T_bol_- T_nadir_	-36 [-45 - -28]	-52 [-63 - -37]	-42 [-49 - -33]	-46 [-61 - -36]	0.040
Δ SAP	T_base_-T_post-bol_	-10 [-21 - 1]	-2 [-10 - 4]	-8 [-15 - -1]	-2 [-7 - 7]	0.074
	T_base_-T_3min_	-27 [-42 - -17]	-31 [-48 - -21]	-32 [-58 - -24]	-22 [-57 - -13]	0.574
	T_base_-T_nadir_	-64 [-74 - -47]	-69 [-93 - -55]	-60 [-82 - -49]	-70 [-86 - -54]	0.452
	T_post-bol_- T_3min_	-15 [-37 - -3]	-32 [-52 - -16]	-30 [-58 - -10]	-28 [-53 - -13]	0.277
	T_post-bol_- T_nadir_	-53 [-66 - -40]	-65 [-92 - -48]	-51 [-77 - -43]	-66 [-87 - -50]	0.142
MAP differential	T_base_-T_post-bol_	0.20 [0.40 -0.09]	0.05 [0.23-0.10]	0.15 [0.27-0.00]	0.0 [0.15-0.10]	0.284
	T_base_-T_3min_	0.08 [0.13-0.03]	0.10 [0.16-0.07]	0.14 [0.16-0.08]	0.08 [0.16-0.05]	0.302
	T_base_-T_nadir_	0.09 [0.11-0.06]	0.10 [0.14-0.08]	0.09 [0.13-0.07]	0.08 [0.12-0.07]	0.445
	T_post-bol_- T_3min_	0.07 [0.13-0.01]	0.11 [0.17-0.07]	0.14 [0.20-0.05]	0.09 [0.19-0.06]	0.317
	T_post-bol_- T_nadir_	0.08 [0.10-0.05]	0.10 [0.15-0.08]	0.09 [0.14-0.06]	0.08 [0.12-0.06]	0.262
SAP differential	T_base_-T_post-bol_	0.31 [0.52-0.03]	0.06 [0.31-0.12]	0.19 [0.41-0.02]	0.05 [0.16-0.19]	0.070
	T_base_-T_3min_	0.13 [0.20-0.08]	0.15 [0.22-0.10]	0.15 [0.26-0.11]	0.10 [0.27-0.06]	0.648
	T_base_-T_nadir_	0.13 [0.19-0.10]	0.13 [0.22-0.10]	0.13 [0.19-0.09]	0.12 [0.17-0.09]	0.829
	T_post-bol_- T_3min_	0.08 [0.21-0.01]	0.16 [0.28-0.10]	0.17 [0.32-0.05]	0.15 [0.29-0.08]	0.288
	T_post-bol_- T_nadir_	0.10 [0.16-0.07]	0.14 [0.26-0.10]	0.13 [0.22-0.08]	0.12 [0.17-0.10]	0.698

As shown in Table 4, heart rate at T_3min_ was significantly higher in group A compared to group C (p = < 0.001) and group D (p = < 0.001), which was also the case for group B, respectively *p* = 0.002 and p = < 0.001. At T_nadir_, group A showed a significant higher heart rate when compared to group C (*p* = 0.009) and group D (p = < 0.001). Heart rate was also higher in Group B compared to group D (*p* = 0.005).

**Table 4:** Median absolute values of heart rate, in beats per minute, and BIS at T_baseline_, T_bolus_, T_3min_ and T_nadir_ with interquartile ranges. At T_3min_ heart rate for groups a and B differed significantly from group C and D. At T_nadir_ heart rate for group a differed significantly from groups C and D, group B differed significantly from group D. At T_3min_ and T_nadir_ for BIS all groups differed significantly from each other

		Group A	Group B	Group C	Group D	*p*-value
Heart rate	T_baseline_	72 [62-78]	84 [69-97]	75 [71-89]	73 [59-84]	0.071
	T_bolus_	80 [67-90]	89 [78-97]	82 [74-89]	76 [58-84]	0.068
	T_3min_	72 [66-80]	73 [65-82]	61 [57-71]	59 [54-67]	<0.001*
	T_nadir_	67 [60-73]	67 [58-75]	59 [54-63]	58 [55-62]	0.002*
BIS	T_baseline_	91 [86-96]	91 [86.5-93]	92.5 [89.25-97]	92 [88-96]	0.622
	T_bolus_	93 [89-97]	94 [85-97]	96 [90.5-97]	95 [88-97]	0.532
	T_3min_	44 [31-57]	67 [53.75-77]	71 [66-83]	83 [74-87]	< 0.001*
	T_nadir_	25 [23-35]	38.5 [31.25-54.25]	57 [47-62.5]	72 [63-78]	< 0.001*

BIS did not differ at T_baseline_ and T_post−bolus_ between groups (Table 4). But at T_3min_ mean BIS tend towards an inverse correlation with the Ce_T, PROP_ targets, although this difference was not statistically significant between groups B and C (*p* = 0.141), and between groups C and D (*p* = 0.06). At T_nadir_ all BIS values were significantly different between all groups.

## Discussion

In this study, we evaluated the immediate haemodynamic changes evoked by induction of general anaesthesia using different combinations of Ce_T, PROP_ and Ce_T, REMI_, all yielding an equipotent PTOL of 90% (as predicted by the Bouillon interaction model). We found that the use of either a low Ce_T, PROP_ in combination with a high Ce_T, REMI_ or vice versa, did result in some statistically significant differences between groups, although the clinical relevance of these small differences could be questioned. As such, our observations could not identify a preferable combination of Ce_T, PROP_ and Ce_T, REMI_, all derived from the same predicted PTOL 90% isobole, that yields more favourable hemodynamic conditions compared to the other drug combination.

A statistically significant difference was observed at T_post−bolus_ (the timepoint directly after the medication bolus) in MAP and SAP between groups A (Ce_T PROP_ 8.6 µg mL^− 1^, Ce_T REMI_ 1.0 ng mL^− 1^) and D (Ce_T PROP_ 2.0 µg mL^− 1^, Ce_T REMI_ 8 ng mL^− 1^), but this difference disappeared at later timepoints. The peak effect of both drugs is expected to take place respectively between 5 and 10 min after the start of infusion for propofol [4] and between 1 and 2 min for remifentanil [16], both being well after T_post−bolus_. This has also been observed in this study where, except for five cases, T_nadir_ always occurred between 3 and 10 min after the start of the infusion.

We also compared heart rates between groups. At T_3min_ and T_nadir_ a statistically significant lower heart rate was observed in groups C and D, the groups which received a relatively high dosage of remifentanil. As a lower heart rate is a common side effect of remifentanil, a lower heart rate in groups C and D was expected.

We used BIS as a reflection of the hypnotic cerebral effect evoked by the administered drug combination, as the hypnotic effect is also an important factor during induction of general anaesthesia. A sufficient hypnotic effect would prevent possible awareness during induction and airway management. Furthermore, it could prevent the physiological response (heart rate and blood pressure fluctuations) to painful airway management manoeuvres and therefore lead to less haemodynamic instability. In our study, patients who received relatively high dosages of propofol showed lower BIS values according to expectations. As the Bouillon model predicts an equal probability of tolerance to a noxious stimulus; our findings are in agreement to previous claims that the BIS has a low predictive performance for a movement response after a noxious stimulus [17]. As a sufficiently powered validation study is still lacking to test the accuracy of the PTOL predictions of the Bouillon model, it remains somewhat speculative whether these patients have reached a real equipotent probability of tolerating laryngoscopy. However, our methodology used every clinically available technology to approach that goal of equipotency to the best of our abilities.

Our findings are not supporting the common belief that high target concentrations of opioids and lower target concentrations of hypnotics during induction will lead to less haemodynamic changes and a lower incidence of post-induction hypotension. This belief is most common in cardiac anaesthesia and probably stems from the period when high-dose opioid techniques during cardiac anaesthesia were favoured [18, 19]. However, a recent meta-analysis in patients having cardiac surgery showed no difference in the use of vasopressors between high- and low-opioid groups [19]. Another recent study demonstrated no statistically significant difference in haemodynamic changes when using different remifentanil doses [20]. Our study endorses these recent findings and further challenges the belief that high opioid anaesthesia equals a lower incidence of post-induction hypotension.

### Clinical interpretation

About one third of intraoperative hypotensive events occur between induction of general anaesthesia and surgical incision [2], the so-called ‘valley of no surgery’. This is the consequence of the administration of anaesthetic drugs in “surgical” doses, and other factors, such as the institution of positive pressure mechanical ventilation. In this unique dataset we compared, on a population level, four effect site concentration combinations from the PTOL = 90 isobole from the Bouillon model. In these specific combinations we were not able to identify an optimal combination as blood pressure changes, and therefore the occurrence of post-induction hypotension, was comparable between groups. To find the optimal combination of effect-site concentrations more data points should be gathered and should be compared on a response surface interaction curve, rather than on a single isobole. A possible approach could be to apply the ‘well-being model’ to quantify not only the desired effects but simultaneously incorporate the undesired side effects of each tested drug combination [21]. Future research could also investigate Ce_T, PROP,_ and Ce_T, REMI_ combinations while performing different induction techniques (for example a rapid sequence induction) or could focus on different populations who are more prone to hypotension.

### Strengths and limitations

A relevant limitation of this study is the inclusion of healthy patients (ASA I to ASA III) without cardiac diseases who underwent neurosurgical or maxillofacial surgical procedures. Therefore, we cannot directly extrapolate these findings to e.g., patients having cardiac surgery. However, the advantage of using a homogenous inclusion of patients is that differences in MAP or SAP can be better correlated to the difference in drug combinations. Another limiting factor is that all data after ephedrine administration were excluded, this may have resulted in an unavoidable but clinically relevant loss of data. The most important limitation of this study are linked to the limitations of the Bouillon model and the absence of a well powered confirmation of its predictive accuracy. However, as the Bouillon model can be used in clinical practice to identify four different effect site concentration combinations with a theoretical equipotency for PTOL, our findings could be reproduced and remain clinical relevant. In order to identify favourable combinations of propofol and remifentanil over a wider range, further research such as described in the “well being model” [22] is required but this will expose a much larger number of patients to study risks. We therefore found this secondary analysis sufficiently informative to explore the induction phase more closely as all groups are related to a single isobole.

A strength is the usage of a volume clamp method (Nexfin) to measure blood pressure, some studies suggest that ABP measured using a volume clamp method may even more closely resemble invasively determined ABP measurements than NIBP [23, 24]. Moreover, the usage of a volume clamp method allowed a continuous assessment of ABP, including more subtle temporal changes in ABP between groups. Another strength is the use of target controlled infusion TCI pumps to reach the desired Ce_T, PROP_ and Ce_T, REMI_ as this increases the reproducibility and external validity of our findings .

## Conclusion

We did not observe a clinically relevant difference in magnitude nor in incidence of postinduction hypotension caused by the usage of four different equipotent Ce_T, PROP_ and Ce_T, REMI_ combinations during induction up until the first eleven minutes of anaesthesia in a population of ASA 1–3 patients undergoing neuro- or maxillofacial surgery. As such, our observations do not support the common belief that the administration of a combination of a low-dose hypnotic together with a high-dose opioid, would provide more favourable haemodynamic conditions compared to an opposite equipotent combination of drugs.

## Data Availability

No datasets were generated or analysed during the current study.
